# Study on the targeted therapy of oral squamous cell carcinoma with a plasmid expressing PE38KDEL toxin under control of the SERPINB3 promoter

**DOI:** 10.1002/cam4.2880

**Published:** 2020-02-03

**Authors:** Jiang Wu, Qiong Guo, Guoliang Zhang, Liying Zhao, Yvguang Lv, Jiaqi Wang, Jiguang Liu, Wei Shi

**Affiliations:** ^1^ School of Stomatology Jiamusi University Jiamusi P.R. China; ^2^ Key Laboratory for Molecular Enzymology & Engineering the Ministry of Education Jilin University Changchun P.R. China; ^3^ College of Pharmacy Jiamusi University Jiamusi P.R. China

**Keywords:** oral squamous cell carcinoma, PE38KDEL toxin, SERPINB3 promoter, targeted therapy

## Abstract

Oral squamous cell carcinoma (OSCC) has a poor prognosis and a high risk of recurrence. To improve the efficacy of OSCC therapy, it is of great significance to explore gene therapy for OSCC. The use of specific genes to regulate the targeted expression of suicide genes is a hot topic in gene therapy for cancer. The SERPINB3 gene is highly active in squamous cell carcinoma, but nearly undetectable or present at a low level in normal tissues. This specificity suggests that the SERPINB3 promoter can be used for targeted OSCC therapy. *Pseudomonas aeruginosa* secretes PE38KDEL, an exotoxin derivative, as a suicide gene used in gene therapy. A SERPINB3 promoter‐mediated PE38KDEL expression vector was created. The SERPINB3 gene expression was tested in different cell lines by RT‐qPCR and Western blotting, and the SERPINB3 promoter activity was detected by luciferase assay. The SERPINB3 promoter was more active in the TCA8113 cell line than in the other cell lines. The target therapeutic potential of the toxin vector pSERPINB3‐PE38KDEL was tested in the SERPINB3‐positive TCA8113 cell line, the SERPINB3‐negative MG63 cell line, and normal L02 cell line. The SERPINB3 gene was expressed at a high level in TCA8113 cells but a low level in MG63 and L02 cells. Transfection of the pSERPINB3‐PE38KDEL plasmid effectively inhibited the proliferation and invasion of TCA8113 cells and induced cell apoptosis, but no significant damage to MG63 and L02 cells was observed. The results of in vitro experiments indicated that the pSERPINB3‐PE38KDEL plasmid could be a promising strategy for targeted OSCC gene therapy.

## Graphical abstract

A SERPINB3 promoter‐mediated PE38KDEL expression new vector was created. The SERPINB3 promoter was more active in the TCA8113 cell line (tongue squamous cell carcinoma) than in the other cell lines. The results of in vitro experiments indicated that the pSERPINB3‐PE38KDEL plasmid could be a promising strategy for targeted OSCC gene therapy.

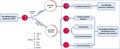

## 1 INTRODUCTION

At present, oral cancer is a major public health problem worldwide.^1^, ^2^ Oral squamous cell carcinoma (OSCC), the most common type of oral cancer, often involves prominent regional and distant lymph node metastases and seriously affects the patient's quality of life.^3^, ^4^, ^5^ The conventional treatments for OSCC are combinations of surgery, radiotherapy, and chemotherapy.^6^, ^7^ Because of the high risk of OSCC recurrence and its poor prognosis, the search for timely and effective clinical treatments for OSCC has become a popular topic for stomatologists.

In recent years, the idea of using gene therapy as a modality in the treatment of diseases other than genetically inherited, monogenic disorders has become established, which is more obvious in the field of oncology.^8^ The use of suicide gene targeting to induce cancer cell death is one of the means of gene therapy for cancer.^9^ Therefore, the targeting of suicide genes has become a hot topic in cancer gene therapy.

The SERPINB3 gene is highly active in squamous cell carcinoma but nearly undetectable or expressed at a low level in normal tissues.^10^ Studies on the targeted treatment of OSCC with the SERPINB3 gene, which regulates the PE38KDEL toxin gene, have not been reported.

*Pseudomonas aeruginosa* exotoxin (PE) is a nonspecific bacterial toxin widely used in tumor therapy.^11^ Its derivative, PE38KDEL, exhibits strong cytotoxicity and low immunogenicity.^12^, ^13^ Therefore, we selected PE38KDEL as the suicide gene for our study.

In the present study, we took advantage of the specific expression of the SERPINB3 gene in squamous cell carcinoma and constructed a pSERPINB3‐PE38KDEL toxin plasmid containing the SERPINB3 gene fragment as promoter by recombinant DNA technology. The specificity and targeted inhibition of the plasmid in the treatment of OSCC were studied by using molecular biological techniques in vitro.

## 2 MATERIALS AND METHODS

### 2.1 Cell culture

This study used the TCA8113 (tongue squamous cell carcinoma), MG63 (osteosarcoma), Eca‐109 (esophageal cancer), HeLa (endocervical adenocarcinoma), MCF‐7 (breast cancer) human cancer cell lines, and the L02 (spontaneously immortalized hepatic cells) normal cell line. The cells were cultured in Dulbecco's modified Eagle's Medium (DMEM) containing 10% fetal bovine serum (FBS) (GibcoBRL), 100 U/mL penicillin, and 100 µg/mL streptomycin at 37°C in a humidified atmosphere containing 5% CO_2_. These cell lines were provided by Prof. Wei Shi (Key Laboratory for Molecular Enzymology & Engineering, the Ministry of Education, provided by Jilin University, China).

### 2.2 Determination of SERPINB3 gene expression in different human cell lines

#### 2.2.1 Western blotting analysis

Total proteins were extracted using a Mammalian Total Protein Extraction kit (Trans) according to the manufacture's introduction, and protein concentrations were determined with the BCA method. The proteins were separated by 12.5% SDS‐PAGE and transferred to PVDF membranes. Then, the transblotted membranes were blocked for 2 hours at room temperature and probed with the corresponding primary antibody overnight at 4°C. After three washes, the membranes were incubated with secondary antibody for 1 hour. Following another three washes, ELC Western Blotting Detection reagents (Trans) and an automatic chemiluminescence image analysis system (Tanon) were used for chemiluminescence detection. This assay was performed in triplicate.

#### 2.2.2 Real‐time fluorescence quantitative PCR

Total RNA was isolated from cells according to the instructions of a TaKaRa Mini BEST Universal RNA Extraction Kit, and the primer sequences used were as follows: sense: 5'‐GGTTACAGAGGAGGGAGCAGAA‐3' and antisense: 5'‐GGGTGATTACAATGGAACTCTTCA‐3'. The amplification was monitored on an ABI Prism 7500 real‐time PCR apparatus (Applied Biosystems) using SYBR Green detection chemistry (TaKaRa). The cycling conditions were as follows: 95°C for 30 seconds followed by 40 cycles of 95°C for 5 seconds and 60°C for 34 seconds. Analysis of the relative fold change in gene expression was performed with the comparative cycle threshold method (2^−ΔΔCt^). All samples were assessed in triplicate.

### 2.3 Construction of plasmids

The luciferase gene reporter constructs were built from the pGL3‐Basic vector, which lacks both promoter and enhancer sequences. The pSERPINB3‐Basic plasmid contains a reporter gene under control of the human SERPINB3 promoter region from nucleotides −1317 to +676 (Ensembl: ENSG00000057149). The promoter was amplified by DNA polymerase chain reaction (sense: 5′‐CCTAGCTAGCGATTAAATGGCCTTGGACAACAACC‐3′ and antisense: 5′‐CATGCCATGGTGGCGGTGAACTCGATGTGATCTGGAACTCC‐3′) and subcloned into NheI and NcoI sites of the pGL3‐Basic vector. The Luciferase gene from the pSERPINB3‐Basic vector was replaced with the PE38KDEL gene to generate the pSERPINB3‐PE38KDEL plasmid. These plasmids were transformed into *E coli* DH5α and confirmed by enzyme digestion and Sanger sequencing analysis.

### 2.4 In vitro transfection

Approximately 1.5 × 10^5^‐2.0 × 10^5^ cells per well were seeded on 6‐well plates. After 24 hours, the cells were prepared for transfection. PEI (C_202_H_505_N_101_) transfection reagent was added to 2 µg of the DNA construct and incubated for 30 minutes in 0.5 mL of serum‐free medium. Following incubation, the DNA‐polycation mixture was added to every well. After 4 hours, the transfected cells were fed 2 mL of growth medium containing 10% FBS.

### 2.5 Activity of the SERPINB3 promoter

The TCA8113, MG63, Eca‐109, HeLa, MCF‐7, and L02 cell lines were seed in 6‐well plates at a density of 2.0 × 10^5^ cells per well and incubated at 37°C for 16 hours before transfection. Then, the cells were transfected with different plasmids (pGL3‐Basic or pSERPINB3‐Basic), and cells transfected with only PEI served as the control. After 48 hours of transfection, the luciferase activity was determined using a Luciferase Assay Kit (Promega). After normalization of the protein content, the expression and activity of luciferase in each group was determined and indirectly reflected the activity of the SERPINB3 promoter in the different cell lines.

### 2.6 Protein synthesis inhibition assay

To account for differences in the experimental results due to differences in transfection efficiency and experimental errors, the TCA8113, MG63, Eca‐109, HeLa, MCF‐7, and L02 cell lines were cotransfected with different amounts (0 µg, 0.025 µg, 0.05 µg, or 0.1 µg) of the indicated plasmids (pGL3‐Basic, pcDNA‐PE38KDEL, or pSERPINB3‐PE38KDEL) and 2 µg of the pGL3‐Control plasmid (containing the SV40 promoter and luciferase cDNA). Cells transfected with the pGL3‐Basic plasmid served as a negative control group, those transfected with the pcDNA‐PE38KDEL plasmid served as a positive control group, and those transfected with the pSERPINB3‐PE38KDEL plasmid served as an experimental group. After 48 hours of cotransfection, the cells were harvested, and the luciferase activity was detected as described above. Because a reduction in luciferase activity can reflect protein synthesis inhibition due to the plasmid, luciferase activity was used to measure the toxicity of the plasmid. This assay was performed in triplicate.

### 2.7 Cell apoptosis and cell cycle arrest assays

The TCA8113, MG63, and L02 cell lines were transfected with different plasmids (pGL3‐Basic or pSERPINB3‐PE38KDEL). The cells were seeded in 6‐well plates at density of 2.0 × 10^5^ cells per well, incubated at 37°C for 16 hours, and then subjected to plasmids transfection. For the cell cycle arrest assay, the cells were cultured with serum‐free medium for 48 hours to deplete the retained growth factors and synchronize the cell cycle before transfection. After 48 hours of transfection, the cells were harvested, and cell apoptosis and cell cycle arrest were detected by flow cytometry. For the cell apoptosis assay, an annexin‐V‐fluorescein isothiocyanate (FITC) and propidium iodide (PI) apoptosis detection kit (Best Bio) was used, while for the cell cycle arrest assay, a PI cycle detection kit (Best Bio) was used. These assays were conducted in triplicate.

### 2.8 Cells early apoptotic changes detected by TUNEL assay

The cells were cultured and transfected as described above. Afterward, the cells were collected after plasmid transfection and evaluated using a TUNEL cell apoptosis assay kit (Biyuntian Biotechnology Co., Ltd.) to examine early apoptotic changes by fluorescence microscope as previously established. Because normal or proliferating cells have little DNA breakage and 3'‐OH formation, they are rarely stained. Cells with broken DNA appear brown‐yellow under a fluorescence microscope.

### 2.9 In vitro Transwell invasion assay

Cell invasion assays was conducted using a 24‐well Transwell chamber of with 8 µm pores (Corning). First, Matrigel was diluted at a ratio of 1:8 and used to coat the upper surface of the bottom membrane of the Transwell chamber. The chamber was incubated at 37°C for 30 minutes, the Matrigel was polymerized into a gel, and the basement membrane was hydrated before use. The TCA8113 and MG63 cell lines were cultured and transfected as above. After 24 hours of transfection, 4 × 10^4^ transfected cells were seeded in the upper chamber with 100 µL of serum‐free DMEM and 700 µL of culture medium containing 10% FBS was added to the lower chamber. After 24 hours, cells that invaded the gel matrix were fixed with absolute methanol for 10 minutes, stained with crystal violet, and photographed under a microscope.

### 2.10 Statistical analyses

Statistical analyses were performed using GraphPad Prism 5.0. All data were expressed as the mean ± SEM. Differences with *P* values <.05 were considered significant. Unless indicated, the results shown in the figures were representative. All the experiments were performed at least three times.

## 3 RESULTS

### 3.1 Expression of the SERPINB3 gene in different human cell lines determined by Western blotting and RT‐qPCR

**Figure 1:**
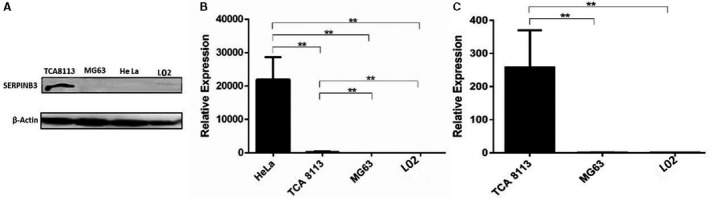
Expression of the SERPINB3 gene in different human cell lines determined by Western blotting and RT‐qPCR. A, SERPINB3 protein expression in TCA8113, MG63, L02, and HeLa cells was determined by Western blotting analysis. B and C, SERPINB3 RNA expression in TCA8113, MG63, L02, and HeLa cells was determined by RT‐qPCR (mean ± SD of three samples, relative to TCA8113, **P < .01)

According to previous reports, the SERPINB3 gene is expressed in mainly squamous cells, and its expression is closely related to cellular differentiation in both normal and malignant squamous cells.^14^, ^15^ To determine the potential of the SERPINB3 gene for the targeted therapy of squamous cell carcinoma, we detected the expression of the SERPINB3 gene in different cell lines (TCA8113, HeLa, MG63, L02) by Western blotting and RT‐qPCR. As shown in Figure 1A,C, SERPINB3 RNA and protein were expressed at low levels in MG63 and L02 cells but were highly expressed in TCA8113 cells. Unexpectedly, as shown in Figure 1B, although the SERPINB3 protein was barely detected in HeLa cells, its RNA was expressed at a high level. This may be due to differences in gene expression at the level of transcription and translation in HeLa cells. Therefore, based on the above results, the SERPINB3 gene was selectively activated in squamous cell carcinoma cells.

### 3.2 Construction of recombinant plasmids

**Figure 2:**
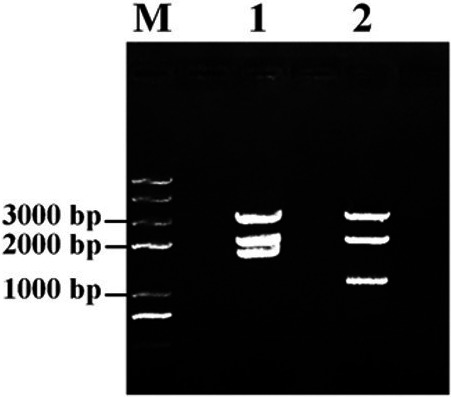
Confirmation of the pSERPINB3‐Basic and pSERPINB3‐PE38KDEL plasmids by restriction endonucleases digestion. M, Trans2K Plus II DNA Ladder; 1, pSERPINB3‐Basic; 2, pSERPINB3‐PE38KDEL

The pSERPINB3‐Basic and pSERPINB3‐PE38KDEL plasmids were constructed and transformed into *E coli* DH5α cells, and the plasmids were then digested with NheI, NcoI, and XbaI restriction endonucleases (NEB). As shown in Figure 2, the SERPINB3 promoter, including restriction endonuclease‐binding sites, was 2004 bp long, the luciferase gene was 1656 bp long, and the PE38KDEL gene was 1049 bp long. Then, the plasmids were further assessed by Sanger sequencing analysis and aligned using NCBI BLAST (data not shown). All the results showed that the recombinant plasmids had been successfully constructed.

### 3.3 Activity of the SERPINB3 promoter

**Figure 3:**
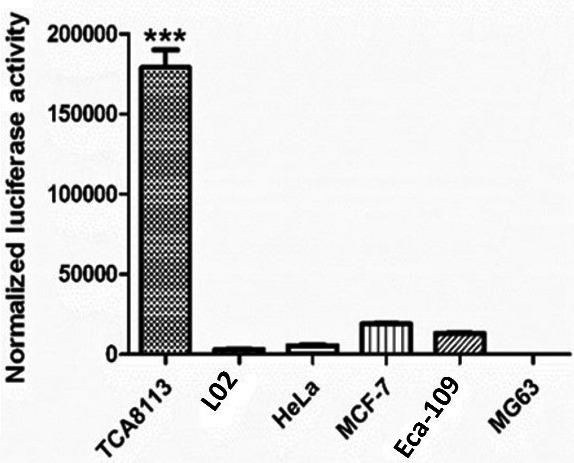
Activity of the SERPINB3 promoter in different cell lines (TCA8113, MG63, Eca‐109, HeLa, MCF‐7, and L02) after 48 h of transfection with the pSERPINB3‐Basic plasmid (mean ± SD of three samples, relative to TCA8113 cells, ***P < .001)

The pSERPINB3‐Basic plasmid was transfected into the TCA8113, MG63, Eca‐109, HeLa, MCF‐7, and L02 cell lines. After 48 hours of transfection, luciferase expressions were detected and then normalized according to the protein content in different cell lines. Figure 3 shows that the luciferase was overexpressed under the control of the SERPINB3 promoter in TCA8113 cells, with a normalized luciferase expression level of 179 037, whereas it was sharply decreased in the MCF‐7, Eca‐109, HeLa, L02, and MG63 cell lines with normalized luciferase expression levels of 19 082.4, 13 244.5, 5487.11, 3151.3, and 11.758, respectively. This result indicated that the SERPINB3 promoter was significantly more active in TCA8113 cells than in other cell lines. Therefore, the TCA8113 cell line considered a SERPINB3‐positive cell line, and the MG63 and L02 cell lines were considered SERPINB3‐negative cell lines.

### 3.4 Inhibition of protein synthesis induced by the pSERPIBN3‐PE38KDEL plasmid

**Figure 4:**
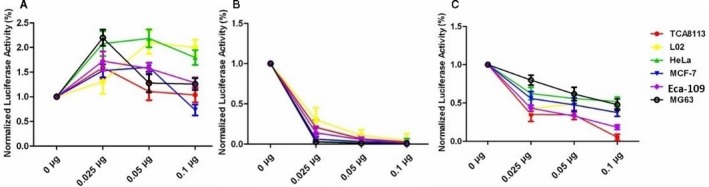
Inhibition of protein synthesis induced by the pSERPIBN3‐PE38KDEL plasmid. A, After 48 h of cotransfected with different quantities (0 µg, 0.025 µg, 0.05 µg, or 0.1 µg) of the negative control plasmid pGL3‐Basic and 2 µg of the pGL3‐Control plasmid (containing SV40 promoter and luciferase cDNA), the luciferase activity was detected in different cell lines (TCA8113, L02, HeLa, MCF‐7, Eca‐109, and MG63). B, After 48 h of cotransfected with different quantities (0 µg, 0.025 µg, 0.05 µg, or 0.1 µg) of the positive control plasmid pcDNA‐PE38KDEL and 2 µg of the pGL3‐Control plasmid, the luciferase activity was detected in different cell lines (TCA8113, L02, HeLa, MCF‐7, Eca‐109, and MG63). C, After 48 h of cotransfected with different quantities of the pSERPINB3‐PE38KDEL plasmid (0 µg, 0.025 µg, 0.05 µg, or 0.1 µg) and 2 µg of the pGL3‐Control plasmid, the luciferase activity was detected in different cell lines (TCA8113, L02, HeLa, MCF‐7, Eca‐109, and MG63)

The PE38KDEL toxin gene can inactive the eukaryotic elongation factor 2 and inhibit cellular protein synthesis. Therefore, a reduction in luciferase activity can be used to reflect the inhibition of protein synthesis by the pSERPINB3‐PE38KDEL plasmid. After 48 hours of cotransfection, curves showing relative luciferase expression were obtained by comparing the luciferase expression in cell lines cotransfected with different amounts of the indicated plasmids with luciferase expression in cell lines cotransfected with pGL3‐Control alone (Figure 4). When 0.1 g of the pGL3‐Basic plasmid was used for transfection, the percentage of luciferase activity in L02, HeLa, Eca‐109, MG63, TCA8113, and MCF‐7 cells was 1.99857%, 1.79688%, 1.28164%, 1.25672%, 1.03583%, and 0.732669%, respectively. Figure 1G shows that the luciferase activity was completely consistent in different cell lines, and when 0.1 g of the pcDNA‐PE38KDEL plasmid was used for transfection, the percentage of luciferase activity in L02, TCA8113, Eca‐109, MCF‐7, HeLa, and MG63 cells was 0.04463%, 0.02666%, 0.01557%, 0.01879%, 0.00519%, and 0.00521%, respectively. Figure 1H shows that the luciferase activity was successively decreased indifferent cell lines, and when 0.1 g of the pSERPINB3‐PE38KDEL plasmid was used for transfection, the percentage of luciferase activity in HeLa, MG63, L02, MCF‐7, Eca‐109, and TCA8113 cells was 0.51828%, 0.47871%, 0.37994%, 0.37436%, 0.18333%, and 0.05174%, respectively. These results showed that among the plasmids tested, the recombinant toxic gene expression plasmid pSERPINB3‐PE38KDEL inhibited the expression of luciferase in TCA8113 cells most strongly, which indirectly indicated that the plasmid strongly inhibits protein synthesis in TCA8113 cells.

### 3.5 Cellular apoptosis and cell cycle arrest assays

**Figure 5:**
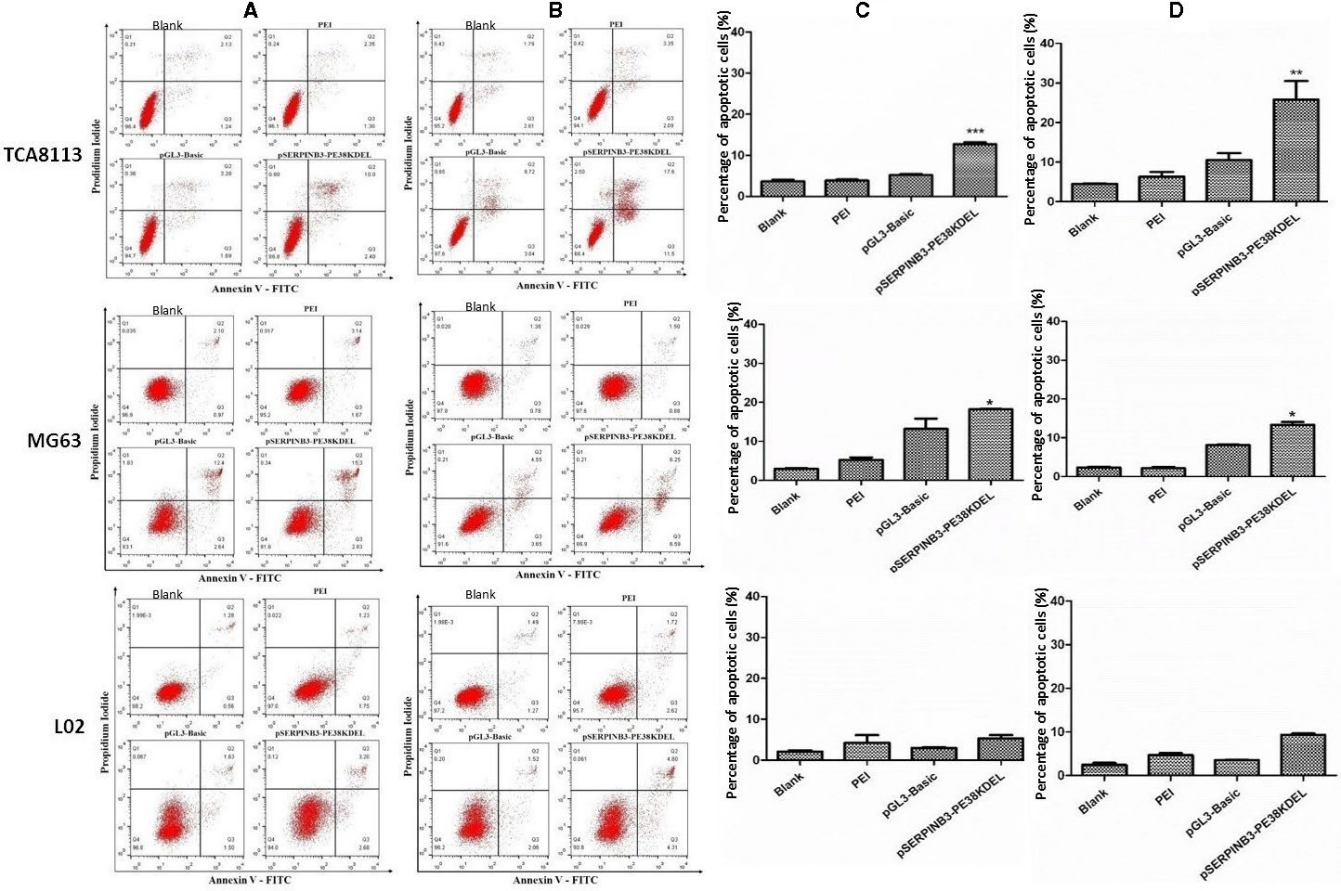
Cellular apoptosis assays after plasmid transfection. A, Cells apoptosis scatter plots after 24 h of transfection. B, Cells apoptosis scatter plots after 48 h of transfection. C, Statistical analysis of the percentages of apoptotic cells after 24 h of transfection. D, Statistical analysis of the percentages of apoptotic cells after 48 h of transfection. TCA8113, MG63, and L02 cells were transfected with different plasmids (pGL3‐Basic or pSERPINB3‐PE38KDEL), and cells transfected with only PEI or cells with no treated (blank) served as the control groups. The results were presented as the mean ± SD of three independent experiments; ***P < .001, **P < .01, *P < .05 vs the control group

**Figure 6:**
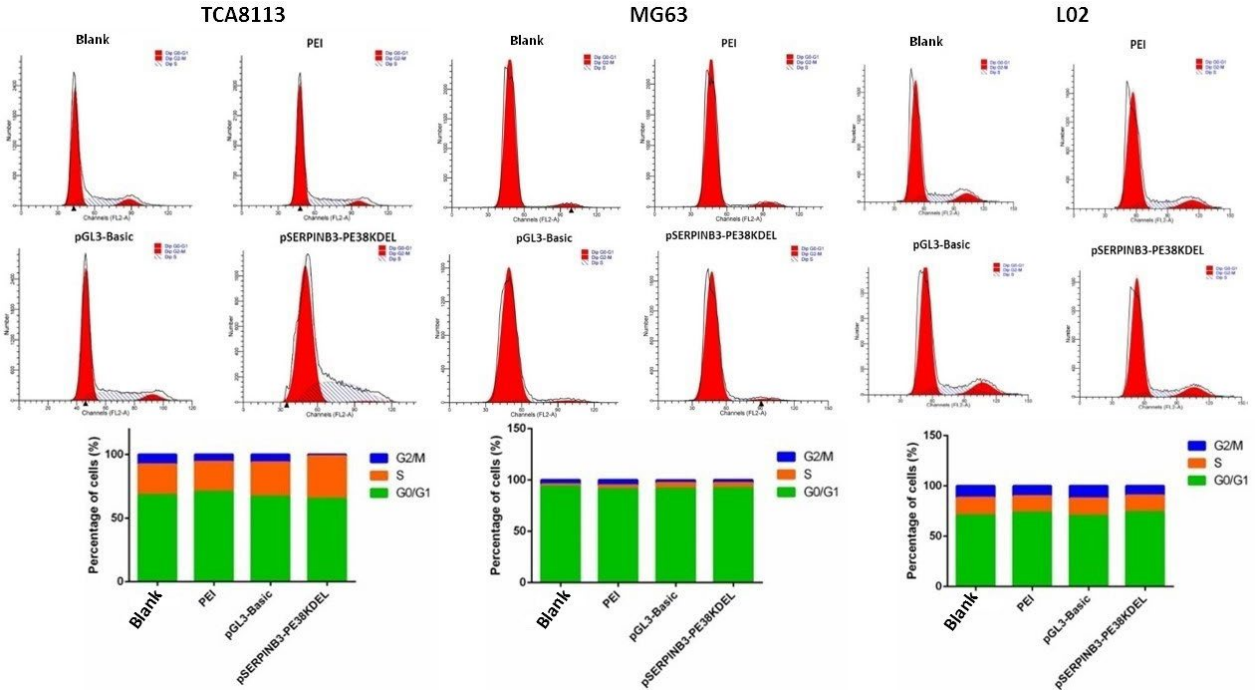
Cell cycle arrest assays after cell cycle synchronization and 48 h of plasmids transfection. TCA8113, MG63, and L02 cells were transfected with different plasmids (pGL3‐Basic or pSERPINB3‐PE38KDEL), and cells transfected with only PEI or cells with no treated (blank) served as the control groups. Histograms were showing the percentage of cells at different phases of the cell cycle and their analysis after 48 h of transfection

This section of the study was carried out to prove that transfection with the pSERPINB3‐PE38KDEL plasmid induced cellular apoptosis and cell cycle arrest by FCM analysis (Figure 5). After 24 hours of transfection, the proportion of apoptotic cells (TCA8113, MG63, and L02 cells) was 12.705%, 18.205%, and 5.33%, respectively. However, after 48 hours of transfection, the proportion of apoptotic cells (TCA8113, MG63, and L02 cells) was 25.78%, 13.355%, and 9.335%, respectively. These results showed that the rate of TCA8113 cell apoptotic necrosis was significantly higher than that of MG63 and L02 cells after 48 hours of transfection. As shown in Figure 6, after cell cycle synchronization and 48 hours of pSERPINB3‐PE38KDEL transfection, 33.58% of TCA8113 cells were in S‐phase, which was a higher percentage than the control group. These results showed that the TCA8113 cell cycle arrested in S phase after pSERPINB3‐PE38KDEL transfection for 48 hours, but there were no significant changes in the cell cycle distribution of L02 and MG63 cells.

### 3.6 Early apoptosis analysis by TUNEL assay

**Figure 7:**
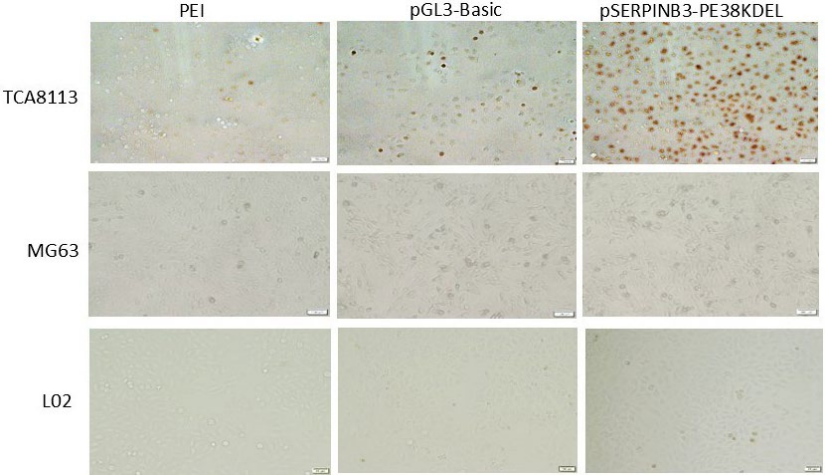
Early apoptosis was determined using TUNEL assays after transfection for 48 h (20 μm). TCA8113, MG63, and L02 cells were transfected with different plasmids (pGL3‐Basic or pSERPINB3‐Basic), and cells transfected with only PEI served as the control group. A few brown‐yellow in L02 and MG63 cells were observed. However, among TCA8113 cells, a mass of brown‐yellow cells indicating DNA fragmentation was observed

After pSERPINB3‐PE38KDEL transfection for 48 hours, as shown in Figure 7, analysis of early apoptotic TCA8113 cells showed that there was a mass of brown‐yellow cells indicating DNA fragmentation, but few brown‐yellow cells indicating early apoptotic cells were observed in MG63 and L02 cells transfected with the different plasmids (pGL3‐Basic or pSERPINB3‐PE38KDEL). These results showed that the pSERPINB3‐PE38KDEL toxin gene plasmid may be an early target to induce apoptosis in OSCC.

### 3.7 In vitro Transwell invasion assay

**Figure 8:**
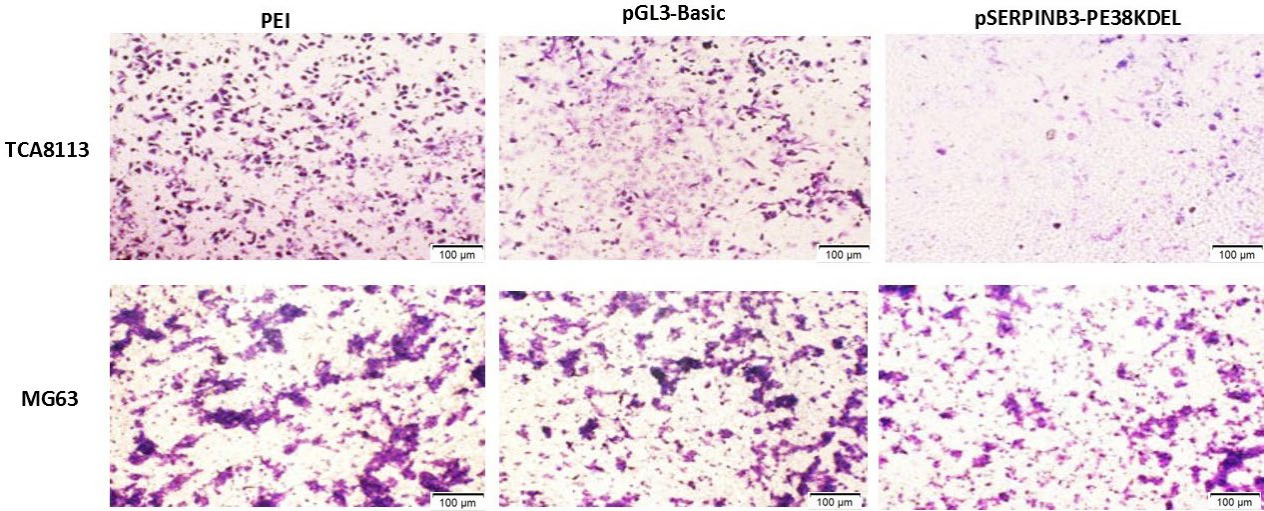
In vitro Transwell invasion assay after plasmids transfection (100 μm). TCA8113 and MG63 cells were transfected with different plasmids (pGL3‐Basic or pSERPINB3‐Basic), and cells transfected with only PEI served as a control. Significantly fewer TCA8113 cells than MG63 cells passed through the gel matrix after pSERPINB3‐PE38KDEL transfection

The Transwell invasion assay was used to judge the influence of toxic plasmids on cell invasion ability, which can be determined according to the number of cells that pass through a gel matrix. The fewer the cells that pass through the gel matrix, the stronger the inhibitory effect of the toxic plasmid on cell invasion ability. Microscope observation showed that the significantly fewer TCA8113 cells than MG63 cells passed through the gel matrix after pSERPINB3‐PE38KDEL transfection, as demonstrated in Figure 8. These results proved that the pSERPINB3‐PE38KDEL plasmid significantly inhibited the invasion of TCA8113 cells.

## 4 DISCUSSION

Gene therapy is a biomedical treatment method based on altering the genetic material of target cells or introducing exogenous genes into target cells to correct or compensate for gene defects and abnormalities with the final goal of treating diseases.^16^ Exogenous genes include suicide gene, tumor suppressor gene, immune gene, silent gene, etc.^17^ Exogenous toxin genes are suicide gene, which have different characteristics from those of conventional chemotherapy drugs, once the suicide gene enters the tumor cell, it continuously utilizes the inherent synthesis mechanism inside the cell to synthesize the toxic protein until the cell dies to improve the durability of tumor therapy. Suicide genes introduced into cells can be directly expressed in the cells, which completely avoids the problems of poor solubility and the lack of stability in the treatment of tumors with exogenous toxin proteins, effectively killing tumor cells, and further reducing the treatment cost.^18^, ^19^

The main purpose of tumor‐targeted gene therapy is to specifically kill tumor cells with no or minimal damage to normal tissues and organs.^20^ Therefore, tumor‐specific genes can be used as promoters to regulate the expression of exogenous toxin genes to achieve tumor‐targeted gene therapy. Tumor‐specific genes are overexpressed in tumor cells and weakly or not expressed in normal cells.

In the 1970s, Kato and Torigoe^21^ isolated squamous cell carcinoma‐specific antigen from cervical squamous cell carcinoma. The antigen is composed of two isomers with almost the same molecular weight, *S*ERPINB3 and SERPINB4, among which SERPINB3 is a serine protease inhibitor.^22^ SERPINB3 is highly expressed in mucous epithelial squamous cell carcinoma of the cervix, esophagus, head and neck, oral cavity, liver, etc, but not expressed or expressed at a low level in normal human squamous cells.^14^, ^15^ The Western blotting and RT‐qPCR results in this study proved that the SERPINB3 gene was expressed at a high level in the SERPINB3‐positive TCA8113 cell line (tongue squamous cell carcinoma), but expressed at a low level in the SERPINB3‐negative cell line MG63 (osteosarcoma) and the normal cell line L02 (spontaneously immortalized hepatic cells). This specificity suggested that the SERPINB3 gene regulated the PE38KDEL toxin‐targeted treatment of OSCC.

The research prospect of suicide gene therapy, in which tumor cells are continuously and effectively inhibited, has attracted much attention.^23^, ^24^ PE is a nonspecific molecular bacterial toxin widely used in tumor therapy. PE derived from *P aeruginosa* consists of a single polypeptide with a relative molecular mass (Mr) of approximately 66 000; PE is composed of 613 amino acids arranged in the following order from the N‐terminus to the C‐terminus: Ia, Ib, II, and III.^25^ Domain Ia (AA1~252) is a membrane transport domain that can transfer active toxin fragments to the cytoplasm, and domain III (AA400~613) is an ADP ribozyme domain. Within the cytoplasm, an ADP‐ribose base is transferred to catalytic EF‐2, slowing chain promotion by EF‐2, which thus inhibits protein synthesis and induces cell death; the function of the Ib domain is less clear.^26^ Domains Ia (△1 − 250) and Ib (△365~380) were deleted, following which PE38 with a smaller Mr was obtained.^27^, ^28^ In addition, when the C‐terminal amino acids of PE were changed from REDLK to KDEL, its transmembrane transport ability and binding activity were greatly improved, enhancing the lethality of the constructed toxin.^29^, ^30^

We used the specific regulation effect of the SERPINB3 gene on the PE38KDEL bacterial toxin, which plays a targeted toxic role in the TCA8113 cell line, to achieve gene targeting in the treatment of tongue squamous cell carcinoma. The limitation of our study was to select only one type of oral squamous cell cancer cell line. In consideration of this problem, we take advantage of the specific expression of the SERPINB3 gene in squamous cell carcinoma, and the rest of our team will continue to study the specificity and targeted inhibition of the pSERPINB3‐PE38KDEL plasmid in the normal oral mucosal epithelial cell lines and other OSCC cell lines. Moreover, future in vivo experiments are needed to further verify the drug toxicity and targeting of the pSERPINB3‐PE38KDEL plasmid and provide a strong theoretical basis for the clinical application of the pSERPINB3‐PE38KDEL plasmid in the targeted treatment of OSCC.

Nonspecific bacterial toxin genes can act as target toxins with the help of cancer‐specific genes to achieve gene therapy in cancer. Therefore, the development of this kind of gene drugs could be widely used in targeted cancer therapy. At present, our team also study the targeted inhibitory effect of PE38KDEL toxin regulated by the specific expression of the CEACAM6 gene in cancer.

## 5 CONCLUSION

This study utilized the specific expression of the SERPINB3 gene in squamous cell carcinoma and constructed the pSERPINB3‐PE38KDEL toxin plasmid with a SERPINB3 gene fragment used as a promoter by recombinant DNA technology. In this study, we used in vitro experiments to prove the targeted inhibitory effect of PE38KDEL toxin regulated by the SERPINB3 gene on OSCC.
